# Supplementary material for: Mitophagy Mediates the Beige to White Transition of Human Primary Subcutaneous Adipocytes Ex Vivo
Source: Pharmaceuticals (Basel). 2022 Mar 17;15(3):363. doi: 10.3390/ph15030363 (PMC8948887; doi:10.3390/ph15030363)
Supplement: Supplementary file 1 [file pharmaceuticals-15-00363-s001.zip › pharmaceuticals-1621052-SI/SUPPLEMENTARY MATERIALS Pharmaceuticals docx.pdf]

SUPPLEMENTARY MATERIALS

Mitophagy Mediates the Beige to White Transition of Human Primary Subcutaneous Adipocytes Ex Vivo

Attila Vámos [1,2,†], Abhirup Shaw [1,2,†], Klára Varga [1,2], István Csomós [3], Gábor Mocsár [3], Zoltán Balajthy [1], Cecília Lányi [4], Zsolt Bacso [3,5], Mária Szatmári-Tóth [1,‡] and Endre Kristóf [1,*,‡]

[1] Laboratory of Cell Biochemistry, Department of Biochemistry and Molecular Biology, Faculty of Medicine, University of Debrecen, H-4032 Debrecen, Hungary; vamos.attila@med.unideb.hu (A.V.); abhirup.shaw@med.unideb.hu (A.S.); klaravarga95@gmail.com (K.V.); balajthy@med.undeb.hu (Z.B.); szatmari-toth.maria@med.unideb.hu (M.S.-T.)
[2] Doctoral School of Molecular Cell and Immune Biology, University of Debrecen, H-4032 Debrecen, Hungary
[3] Department of Biophysics and Cell Biology, Faculty of Medicine, University of Debrecen, H-4032 Debrecen, Hungary; csomos.istvan@med.unideb.hu (I.C.); mocsgab@med.unideb.hu (G.M.); bacso@med.unideb.hu (Z.B.)
[4] Laser Clinic, H-1012 Budapest, Hungary; lezerklinika@gmail.com
[5] Faculty of Pharmacy, University of Debrecen, H-4032 Debrecen, Hungary
* Correspondence: kristof.endre@med.unideb.hu; Tel.: +36-52-416-432
† These authors have contributed equally to this work.
‡ These authors have contributed equally to this work.

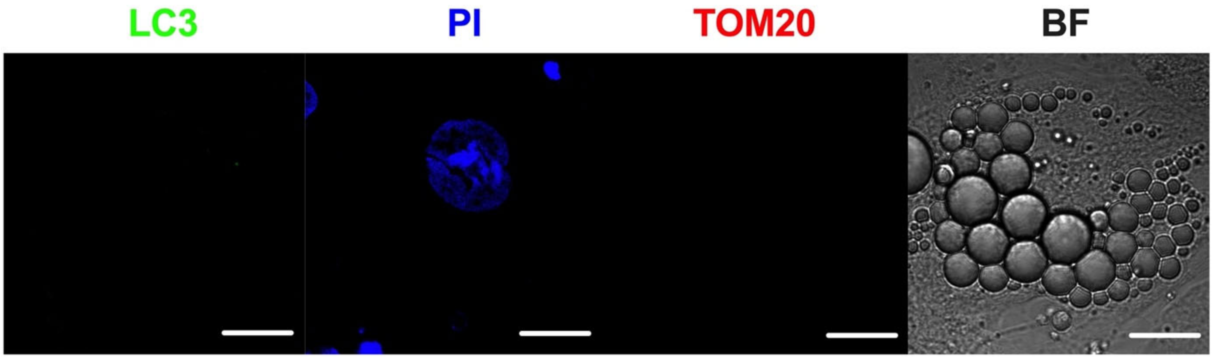

Figure S1: Secondary antibody control images showing the specificity of the antibodies used for LC3 and TOM20 immunostaining. PI labels the nucleus. BF represents brightfield image. Scalebars represent 10μm.

Table S1: Table listing all gene expression assays used in the study

Gene name	Assay ID
UCP1	Hs00222453_m1
PPARGC1A	Hs01016719_m1
GAPDH	Hs99999905_m1
PARK2	Hs01038322_m1
SQSTM1	Hs00177654_m1
OPTN	Hs00184221_m1
NDP52	Hs00977443_m1
BNIP3	Hs00969291_m1
BNIP3L	Hs00188949_m1
FKBP8	Hs01014664_m1
BCL2L13	Hs00209789_m1
FUNDC1	Hs00697693_m1
CIDEA	Hs00154455_m1
LEP	Hs00174877_m1
